# Prospectus of the Savannah Journal of Medicine

**Published:** 1858-04

**Authors:** 


### Prospectus of the Savannah Journal of Medicine.

We have received the prospectus of a Journal to be published under the above title in Savannah, Ga. JURIAH HARRIS, M. D., Professor of Physiology in the Savannah Medical College, and J. S. SULLIVAN, M. D., are to be the editors, and

R. D. Arnold, M. D., Professor of the Principles and Practice of Medicine in the Savannah Med. College, associate editor. Although there are already two medical journals in successful operation in the State, still we are confident that another may be sustained, if the profession can only be induced to regard such an enterprise in its true light. Let us not hear it said that there are already a sufficient number of medical journals in the State, but rather, let us be proud that Georgia, in that as in many other enterprises, is outstripping her sister States. Let the profession come up to the work, and sustain her medical journals, and 'ere long, many of the short-comings which are at present so much deplored, will be seen no more. The above Journal is to be a bi-monthly, of seventy-two octavo pages. From the prospectus we learn that every arrangement has been made for its establishment and continuation. The first number will be issued about the first of May. Terms in advance—single copy $2 00; three copies $5 00; six copies $9 00. We wish the enterprise every success, and place it with pleasure upon our exchange list.

